# The reliability and validity of a developed anxiety scale specific to primary focal hyperhidrosis symptoms

**DOI:** 10.1186/s13030-024-00310-y

**Published:** 2024-06-04

**Authors:** Sayaka Ogawa, Jun Tayama, Hiroyuki Murota, Masakazu Kobayashi, Hirohisa Kinoshita, Seiko Nakamichi

**Affiliations:** 1 Faculty of Humanities, Nagasaki Junshin Catholic University, 235 Mitsuyama-Mach, Nagasaki, 852-8558 Japan; 2 Faculty of Human Sciences, Waseda University, 2-579-15 Mikajima, Tokorozawa, 359-1192 Japan; 3 Department of Dermatology, Nagasaki University, 1-7-1 Sakamoto, Nagasaki, 852-8501 Japan; 4 Health Center, Nagasaki University, 1-14 Bunkyo-Machi, Nagasaki, 852-8521 Japan

**Keywords:** Anxiety, Hyperhidrosis, Scale, Reliability, Validity

## Abstract

**Background:**

Patients with primary focal hyperhidrosis (hyperhidrosis) are known to have higher levels of anxiety induced by sweating than those who do not. However, in hyperhidrosis, no scale has been developed to measure anxiety specific to hyperhidrosis symptoms. Therefore, this study aimed to develop an anxiety scale specific to hyperhidrosis symptoms (ASSHS) and to verify its reliability and validity.

**Methods:**

Based on previous studies on hyperhidrosis and a preliminary survey conducted with 26 university students who met the diagnostic criteria for hyperhidrosis, 40 items that adequately reflected anxiety specific to hyperhidrosis symptoms were obtained. A survey was done to examine the internal consistency and validity of the our developed ASSHS. In total, 1,207 participants (680 male and 527 female; mean age ± standard deviation 18.7 ± 0.9 years) were included. A second survey (re-survey) was conducted three weeks later to verify the reliability. It included 201 participants (85 male and 116 female; mean age ± standard deviation 18.6 ± 0.7 years). The survey items included (1) the diagnostic criteria for hyperhidrosis, (2) our anxiety scale developed for primary focal hyperhidrosis symptoms (ASSHS), (3) Hyperhidrosis Disease Severity Scale (HDSS), (4) State-Trait Anxiety Inventory (STAI), (5) Hospital Anxiety and Depression Scale (HADS), (6) Dermatology Life Quality Index (DLQI), and (7) presence of anxiety induced by sweating.

**Results:**

The results of the factor analysis revealed 10 items with one factor, “anxiety specific to hyperhidrosis symptoms.” The alpha coefficient of the ASSHS was *α* = 0.94. The correlation coefficient between the scores at re-test was *r* = 0.75. A moderate positive correlation was found between the ASSHS, HDSS (*r* = 0.53), and anxiety induced by sweating (*r* = 0.47) (all *p* < 0.001). Additionally, participants with hyperhidrosis symptoms had significantly higher ASSHS scores than did those without hyperhidrosis symptoms (*p* < 0.001). Those with mild/moderate hyperhidrosis and those with severe hyperhidrosis had significantly higher the ASSHS scores than did those without hyperhidrosis (*p* < 0.001).

**Conclusions:**

This scale has sufficient reliability and validity as an instrument to measure anxiety specific to hyperhidrosis symptoms.

## Introduction

Primary focal hyperhidrosis (hyperhidrosis) is a disease characterized by bilateral excessive sweating from localized sites, such as palmar, plantar, axillary, and head/face [1]. Its prevalence was 16.7%, 14.8%, 12.3%, 5.5%, 4.8%, and 4.6% in Poland [2], Shanghai [3], Vancouver [3], Sweden [4], the United States [5], and Germany, respectively [6]. Its overall prevalence was 10.0% in Japan, and the prevalence by sweating site was 5.9%, 3.6%, 2.9%, and 2.3% for the axillary, head/face, palmar, and plantar, respectively [7].

Patients with hyperhidrosis have a decreased quality of life (QOL) due to their symptoms [8]. Several psychological aspects have been reported regarding QOL disorders among patients with hyperhidrosis, including high general anxiety [9, 10]. A retrospective cohort study of 44,484 individuals diagnosed with hyperhidrosis in the United States revealed a 17.4% prevalence of reported anxiety in the past 12 months among 137,451 healthy participants, compared with 27.8% in the hyperhidrosis group [11]. Another study evaluated 197 Brazilian patients with hyperhidrosis for anxiety and depressive symptoms via the Hospital Anxiety and Depression Scale (HADS) questionnaire and found that the percentages of highly anxiety and highly depression in patients with hyperhidrosis were 49.2% and 11.2%, respectively [9]. Among patients with hyperhidrosis, the percentage of those with high anxiety was four times higher than that of patients with high depression [9]. A study used the Generalized Anxiety Disorder (GAD-7) scale to screen 2,017 individuals who visited dermatology outpatient clinics in Canada and China for anxiety symptoms and revealed a 7.5% prevalence of anxiety symptoms in patients without hyperhidrosis compared to a significantly higher 23.1% prevalence in patients with hyperhidrosis [10].

A systematic review of the relation between hyperhidrosis and anxiety reported that the GAD-7, HADS, Trier Inventory for Chronic Stress, Beck Anxiety Inventory, Social Phobia Inventory, Leibowitz Social Anxiety Scale, and Spielberger State Trait Anxiety Inventory were often used to measure anxiety [12]. Anxiety scales used in previous studies on hyperhidrosis measured general anxiety, not anxiety specific to hyperhidrosis symptoms. The Itch Anxiety Scale for Atopic Dermatitis (IAS-AD) was developed for atopic dermatitis, the same skin disease as hyperhidrosis. Furthermore, the IAS-AD measures disease-specific anxiety, which is different from general anxiety [13]. However, no scale has been developed to measure anxiety specific to hyperhidrosis symptoms. If anxiety specific to hyperhidrosis symptoms could be measured, it would lead to a better understanding of the psychological aspects of hyperhidrosis patients and be useful in their treatment.

Only two previous studies on anxiety were limited to hyperhidrosis symptoms [14, 15]. In a study that compared 40 hyperhidrosis patients who sought surgical treatment with 64 patients who sought treatment for social anxiety disorder in the United States, 76% of patients with hyperhidrosis reported increased social anxiety due to sweating [14]. A web-based survey of 1,080 Japanese university students revealed that the odds ratio of anxiety induced by sweating, adjusted for age and sex, was 9.72 (95% Confidence Interval: 5.80–16.27). Therefore, the anxiety induced by sweating was significantly higher among those with hyperhidrosis symptoms than among those without. Additionally, the odds ratio of anxiety was significantly higher in participants with mild/moderate and severe hyperhidrosis than in those without [15]. 

This study aimed to verify the reliability and validity of our developed anxiety scale specific to hyperhidrosis symptoms (ASSHS). We compared anxiety scores specific to hyperhidrosis symptoms and each item according to the presence or absence of hyperhidrosis symptoms, their severity, and sweating site. We also calculated the cutoff scores of the ASSHS.

## Methods

### Development of the ASSHS items

We referred to previous studies related to hyperhidrosis that included diagnostic criteria [16], severity assessment [17], hyperhidrosis’ impact on QOL [8], and the Hyperhidrosis Quality of Life Index [18], to select specific scale items that capture anxiety associated with hyperhidrosis symptoms. Additionally, to understand the actual state of anxiety regarding their symptoms, 26 university students who met the diagnostic criteria and consented to participate in the preliminary survey were asked, “What kind of anxiety do you have due to sweating in your daily life?” Based on the results of the previous studies and a preliminary survey, 40 items that adequately reflected anxiety specific to hyperhidrosis symptoms were obtained.

### Study participants

We asked 2,514 university students to respond to the questionnaire and received 1,223 responses from those who provided consent. Of these, seven participants aged 26 years or older and three participants who answered “other” for sex were excluded. Subsequently, among those who met the diagnostic criteria for hyperhidrosis, participants with sweating sites on the buttocks (two participants), back (two participants), nose (one participant), and back of the neck (one participant) were excluded. In the first survey, 1,207 participants (680 male and 527 female, mean age ± standard deviation 18.7 ± 0.9 years) available for inclusion in the study.

To examine the retest reliability of the ASSHS, a second survey was conducted three weeks after the first survey with 201 students who agreed to participate (85 male and 116 female; mean age ± standard deviation 18.6 ± 0.7 years).

### Study procedure

The study period was from 2021 to 2022. After a university lecture, the researcher explained the study’s purpose, voluntary nature of responses, protection of personal information, and withdrawal of consent, both orally and in writing, and requested cooperation. A link to the questionnaire was provided to the participants, and their consent was obtained after they submitted the questionnaire.

### Measurements

#### Diagnostic criteria for hyperhidrosis [16] and site of high sweating

For the diagnostic criteria for hyperhidrosis, we used the criteria of Hornberger et al. (2004) [16]. Participants were asked, “Have you had excessive localized sweating on the head/face, palmar, plantar, and axillary for more than six months (yes or no) without any apparent cause?” Those who answered “yes” were further asked the following questions: (1) Were you under 25 years old when the symptoms first appeared? (2) Do you have symmetrical sweating? (3) Does the sweating stop when sleeping? (4) Do you have at least one episode of hyperhidrosis per week? (5) Do you have family members with hyperhidrosis? (6) Does the sweating interfere with your daily life? Respondents who answered “yes” to at least two questions were judged as hyperhidrosis-symptomatic.

Regarding their high sweating site, participants were asked, “Which of the following sites causes the most sweating: head/face, palmar, plantar, axillary, or other sites? Please select one item that is applicable.”

#### Provisional ASSHS developed for this study

The 40 items prepared in this study were used as a provisional version of the ASSHS. Participants carefully reviewed the subsequent statements and assigned a numerical value to each item that best represented their situation. Responses were rated on a 5-point scale that ranged from 0 (not at all applicable) to 4 (very applicable).

#### Severity of hyperhidrosis (HDSS) [17]

The Hyperhidrosis Disease Severity Scale (HDSS) of Strutton et al. (2004) [17] was used to determine hyperhidrosis severity. Participants selected one of the four provided options that most closely represented their subjective symptoms and frequency of sweating: “1. My sweating is never noticeable and never interferes with my daily activities,” “2. My sweating is tolerable but sometimes interferes with my daily activities,” “3. My sweating is barely tolerable and frequently interferes with my daily activities,” and “4. My sweating is intolerable and always interferes with my daily activities.” Those who selected 1 and 2 were defined as mild/moderate, and 3 and 4 as severe [19–21].

#### State-trait anxiety inventory (STAI) [22, 23]

The State-Trait Anxiety Inventory (STAI) includes 40 items and two subscales (20 items of State anxiety and 20 items of Trait anxiety). Each item is rated on a 4-point scale from 1 to 4, and higher scores indicate greater anxiety. The total scores range from 20 to 80 points.

#### Hospital anxiety and depression scale (HADS) [24, 25]

The Hospital anxiety and depression scale (HADS) is a 14-item self-administered anxiety and depression scale that includes seven anxiety items (HADS Anxiety) and seven depression items (HADS Depression) [24]. Each item is rated on a 4-point scale from 0 to 3, and higher scores indicate more symptoms of anxiety or depression. The total scores of the two subscales range from 0 to 21 points.

#### Dermatology life quality index (DLQI) [26]

The Dermatology Life Quality Index (DLQI) assesses health-related QOL among individuals with skin diseases and includes 10 items [26]. Each item is rated on a 4-point scale from 0 to 3, and higher scores indicate worse health-related QOL associated with skin diseases [26]. 

#### Presence of anxiety induced by sweating [15]

We the method of Ogawa et al. (2023) [15] to assess the presence of anxiety induced by sweating. Participants were asked, “Do you have anxiety induced by sweating?” and were given the option to choose “yes” or “no.”

### Statistical analysis

An exploratory factor analysis with varimax rotation was conducted on the ASSHS. Items with factor loadings of 0.75 or less were excluded. Subsequently, a confirmatory factor analysis was performed, and the chi-square (*χ*^2^), goodness-of-fit index (GFI), adjusted goodness-of-fit index (AGFI), comparative fit index (CFI), and root mean square error of approximation (RMSEA) values were calculated as goodness-of-fit indices. In general, when performing confirmatory factor analysis, it is preferable to use data obtained from a different participant than the data used in exploratory factor analysis. However, in this study, considering the rarity of data obtained from a specific group of university students during the preferred period for hyperhidrosis, we exceptionally conducted both exploratory factor analysis and confirmatory factor analysis using data obtained from the same participants as the exploratory factor analysis in order to make effective use of the limited data. Cronbach’s alpha coefficient and Pearson’s product-rate correlation coefficient with scores at retest were used to examine reliability. Pearson’s product-rate correlation coefficient and Spearman’s rank correlation coefficient were calculated for validity.

To compare the means of the anxiety scale scores specific to hyperhidrosis symptoms, STAI state anxiety, STAI trait anxiety, HADS anxiety, HADS depression, and DLQI according to the presence of hyperhidrosis symptoms, t-tests and effect sizes (*d*) were calculated. Next, a one-factor analysis of variance (ANOVA) was performed to compare differences in anxiety scale scores specific to hyperhidrosis symptoms, STAI state anxiety, STAI trait anxiety, HADS anxiety, HADS depression, and DLQI by hyperhidrosis symptom severity (no symptoms, mild/moderate, severe). Bonferroni’s multiple comparisons test was performed, and the effect size (*η*^2^) was calculated. A similar analysis was conducted via sweating site (no symptoms, palmar, plantar, axillary, head/face) as an independent variable. Furthermore, cutoff values were calculated by receiver operating characteristic (ROC) curves.

All analyses were conducted using SPSS Statistics version 28 and Amos version 28 (IBM Japan Inc., Tokyo, Japan) and values of *p* < 0.05 (two-tailed) were considered statistically significant.

### Ethical considerations

Regarding ethical considerations, the explanatory document provided to participants stated the study’s purpose, protection of personal information, that cooperation was voluntary, and that no disadvantages would be incurred by refusing to cooperate. We requested in writing that only those who provided their consent could respond to the survey. This study was approved by the ethics committee of the Nagasaki University Graduate School of Biomedical Sciences (approval number: 20042101).

## Results

### Demographic data

The mean (standard deviation) age of participants was 18.7 (0.9) years. Participants with hyperhidrosis symptoms totaled 129 (11%). Regarding hyperhidrosis severity, 1078 (89%) were negative screen for hyperhidrosis, 89 (8%) were mild/moderate, and 40 (3%) were severely symptomatic. Regarding sweating site, 1078 (89%) were negative screen for hyperhidrosis, 56 (5%) had palmer, 13 (1%) plantar, 44 (4%) axillary, and 16 (1%) head/face hyperhidrosis. The mean (standard deviation) scores of participants were 11.8 (10.0) for the ASSHS, 40.3 (10.0) for STAI State anxiety, 45.5 (9.8) for STAI Trait anxiety, 7.6 (3.7) for HADS Anxiety, 5.9 (3.2) for HADS Depression, and 1.9 (3.3) for DLQI.

### Exploratory factor analysis

An exploratory factor analysis was conducted on the 40 items of the ASSHS. The number of factors was estimated to be 1 based on the attenuation of the scree plot. The 30 items with low factor loadings (less than 0.75) were deleted, and 10 items were finally adopted after examining the overlap in item content (Table 1). The factor was named “anxiety specific to hyperhidrosis symptoms.”

**Table 1:** Results of the factor analysis

Item no	Items	Factor loading	*α* coefficient
31	I feel anxious that people will point out my sweat	0.82	0.94
32	I feel anxious that I might get sweat on anything I touch	0.81	
19	I feel anxious that my sweat will wet things	0.77	
30	When I hand over change at a store, I am worried that I might feel uncomfortable due to sweat	0.77	
12	I feel anxious that I am causing discomfort to others by my sweat	0.77	
21	I feel anxious that when I fill out the paperwork, the sweat will wet the paper	0.76	
34	I feel anxious about sweating on the floor when I am barefoot	0.76	
9	I feel anxious about sweating when I go out in public	0.76	
38	I feel anxious that I will not be able to concentrate on work (school) due to sweat	0.76	
29	When I high-five someone, I feel anxious that I may cause discomfort due to sweat	0.75	

### Confirmatory factor analysis

**Fig. 1:**
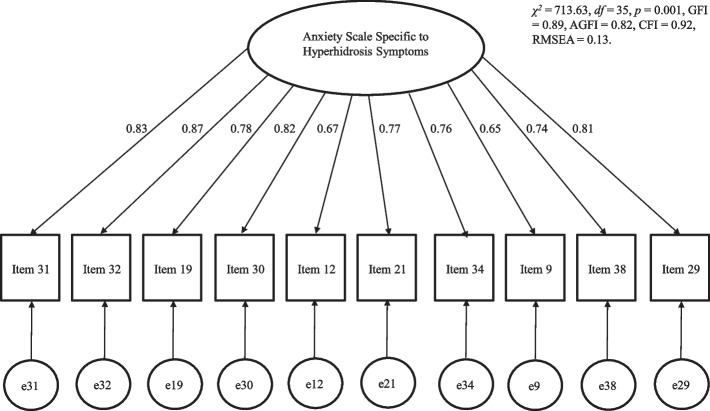
Results of the covariance structural analysis. *Note.* Numbers adjacent to the arrows indicate factor loadings. *Abbreviations.* GFI: goodness-of-fit index, AGFI: adjusted goodness-of-fit index, CFI: comparative fit index, RMSEA: root mean square error of approximation

A confirmatory factor analysis was conducted on the 10-item version of the ASSHS. Consequently, *χ*^2^ = 713.63, degrees of freedom (*df)* = 35,* p* = 0.001, GFI = 0.89, AGFI = 0.82, CFI = 0.92, and RMSEA = 0.13. RMSEA was not a good indicator of model fit. However, the other indicators had acceptable values (Fig. 1).

### Consideration of reliability

The alpha coefficient of the ASSHS was *α* = 0.94 for the first survey (*n* = 1,207). Regarding test–retest reliability, the correlation coefficient between the scores at re-test after a three-week interval was *r* = 0.75 (*p* < 0.001).

### Correlation between the ASSHS and each scale

A moderate positive correlation was found between the ASSHS, HDSS (*r* = 0.53), which measured the severity of hyperhidrosis, and anxiety induced by sweating (*r* = 0.47) (all *p* < 0.001). A weak positive correlation was observed between the ASSHS and STAI State anxiety (*r* = 0.24), STAI Trait anxiety (*r* = 0.30), and HADS Anxiety (*r* = 0.30) (all *p* < 0.001).

### Comparison of the ASSHS and each score based on the presence of hyperhidrosis symptoms

Table 2 shows the results of the comparison of each mean according to the presence or absence of hyperhidrosis symptoms. The ASSHS (*t* (1205) = -13.74, *p* < 0.001, *d* = 1.28), STAI Trait anxiety (*t* (1205) = -4.19, *p* < 0.001, *d* = 0.39), and HADS Anxiety (*t* (1205) = -3.34, *p* < 0.001, *d* = 0.31) were significantly higher in those with hyperhidrosis than in those without. Additionally, the STAI State anxiety (*t* (1205) = -2.13, *p* < 0.05, *d* = 0.20) and the DLQI scores (*t* (1205) = -2.23, *p* < 0.05, *d* = 0.21) were also significantly higher in those with hyperhidrosis than in those without.

**Table 2:** Comparison of each score based on the positive and negative screens for hyperhidrosis

Variables	Negative screen for hyperhidrosis	Positive screen for hyperhidrosis	*t*-value	*df*	*p-*value	Effect size *d*
	(*n* = 1078)	(*n* = 129)				
ASSHS	10.49 ± 9.12	22.41 ± 10.56	-13.74	1205	< 0.001	1.28
STAI State anxiety	40.10 ± 10.02	42.09 ± 9.88	-2.13	1205	0.033	0.20
STAI Trait anxiety	45.14 ± 9.66	48.92 ± 10.01	-4.19	1205	< 0.001	0.39
HADS Anxiety	7.49 ± 3.70	8.64 ± 3.69	-3.34	1205	< 0.001	0.31
HADS Depression	5.89 ± 3.19	5.64 ± 2.94	0.83	1205	0.406	0.08
DLQI	1.79 ± 3.28	2.48 ± 3.44	-2.23	1205	0.025	0.21

### Comparison of the ASSHS and each score based on hyperhidrosis severity

Table 3 shows the results of the comparison of each mean according to the severity of hyperhidrosis symptoms. Results of the one-factor ANOVA showed a main effect of hyperhidrosis severity on ASSHS scores (*F* (2,1204) = 104.96, *p* < 0.001, *η*^2^ = 0.15). Bonferroni’s multiple comparisons revealed that those with mild/moderate and severe hyperhidrosis had significantly higher ASSHS scores than did those without (all *p* < 0.001). Additionally, those with severe hyperhidrosis had significantly higher ASSHS scores than did those with mild/moderate hyperhidrosis (*p* < 0.001).

**Table 3:** Comparison of each score based on hyperhidrosis severity

Variables	Negative screen for hyperhidrosis (0)	Mild, Moderate (1)	Severe (2)	*F*	*df*	*p-*value	Results of multiple comparisons	Effect size *η*^2^
	(*n* = 1078)	(*n* = 89)	(*n* = 40)					
ASSHS	10.49 ± 9.16	20.07 ± 10.43	27.63 ± 8.94	104.96	2, 1204	< 0.001	0 < 1 < 2^a^	0.15
STAI State anxiety	40.10 ± 10.02	41.80 ± 9.63	42.73 ± 10.50	2.39	2, 1204	0.092	-	0.00
STAI Trait anxiety	45.14 ± 9.66	48.48 ± 9.77	49.90 ± 10.59	9.08	2, 1204	< 0.001	0 < 1, 2^b^	0.02
HADS Anxiety	7.49 ± 3.70	8.40 ± 3.78	9.18 ± 3.47	6.15	2, 1204	0.002	0 < 2^c^	0.01
HADS Depression	5.89 ± 3.19	5.56 ± 2.68	5.86 ± 3.17	0.44	2, 1204	0.644	-	0.00
DLQI	1.79 ± 3.28	2.34 ± 3.50	2.80 ± 3.33	2.79	2, 1204	0.062	-	0.01

*Note*: ^a^Results compared to 0 by Bonferroni’s multiple comparisons. *p* < 0.001; ^b^Results compared to 0 by Bonferroni’s multiple comparisons. *p* < 0.01; ^c^Results compared to 0 by Bonferroni’s multiple comparisons. *p* < 0.05

A main effect of hyperhidrosis severity on the STAI Trait anxiety (*F* (2,1204) = 9.08, *p* < 0.001, *η*^2^ = 0.02) was observed. Furthermore, Bonferroni’s multiple comparisons showed that the STAI Trait anxiety was significantly higher in those with mild/moderate and severe hyperhidrosis than in those without (all *p* < 0.01). A main effect of hyperhidrosis severity on the mean HADS Anxiety (*F* (2,1204) = 6.15, *p* < 0.01, *η*^2^ = 0.01) was also observed. Bonferroni’s multiple comparisons showed that those with severe hyperhidrosis had significantly higher HADS Anxiety scores than did those with no symptoms (*p* < 0.05).

### Comparison of the ASSHS and each score based on site of sweating

Table 4 shows the results of the comparison of each mean value based on the sweating site. Results of the one-factor ANOVA revealed a main effect for the ASSHS scores (*F* (4,1202) = 49.03, *p* < 0.001, *η*^2^ = 0.14). Bonferroni’s multiple comparisons revealed that participants with palmar, plantar, axillary, and head/face hyperhidrosis had significantly higher the ASSHS scores than did those without (all *p* < 0.001). A main effect was observed for the STAI Trait anxiety (*F* (4,1202) = 4.52, *p* < 0.001, *η*^2^ = 0.02) and HADS Anxiety (*F* (4,1202) = 3.45, *p* < 0.01, *η*^2^ = 0.01) based on the site of sweating. Additionally, Bonferroni’s multiple comparisons revealed that participants with axillary hyperhidrosis had significantly higher STAI Trait anxiety (*p* < 0.05) scores than did those without axillary hyperhidrosis.

**Table 4:** Comparison of each score based on site of high sweating

Variables	Negative screen for hyperhidrosis (0)	Palmar (1)	Plantar (2)	Axillary (3)	Head/Face (4)	*F*	*df*	*p-*value	Multiple comparisons	Effect size *η*^2^
	(*n* = 1078)	(*n* = 56)	(*n* = 13)	(*n* = 44)	(*n* = 16)					
ASSHS	10.49 ± 9.16	24.21 ± 10.46	24.69 ± 11.13	19.68 ± 10.78	21.75 ± 8.87	49.03	4, 1202	< 0.001	0 < 1, 2, 3, 4^a^	0.14
STAI State anxiety	40.10 ± 10.02	42.75 ± 9.92	40.15 ± 8.98	42.18 ± 9.53	41.06 ± 11.85	1.36	4, 1202	0.246	-	0.01
STAI Trait anxiety	45.14 ± 9.66	48.84 ± 9.08	47.23 ± 8.25	49.41 ± 11.57	49.25 ± 10.54	4.52	4, 1202	< 0.001	0 < 3^b^	0.02
HADS Anxiety	7.49 ± 3.70	8.30 ± 3.09	9.08 ± 5.07	8.48 ± 3.73	9.94 ± 4.28	3.45	4, 1202	0.008	-	0.01
HADS Depression	5.89 ± 3.19	5.50 ± 2.65	4.62 ± 1.94	6.18 ± 3.45	5.50 ± 3.01	0.87	4, 1202	0.481	-	0.00
DLQI	1.79 ± 3.28	2.48 ± 3.22	3.77 ± 4.19	1.98 ± 3.40	2.81 ± 3.69	2.05	4, 1202	0.085	-	0.01

*Note:* ^a^Results compared to 0 by Bonferroni’s multiple comparisons. *p* < 0.001; ^b^ Results compared to 0 by Bonferroni’s multiple comparisons. *p* < 0.05

### ROC curve

**Fig. 2:**
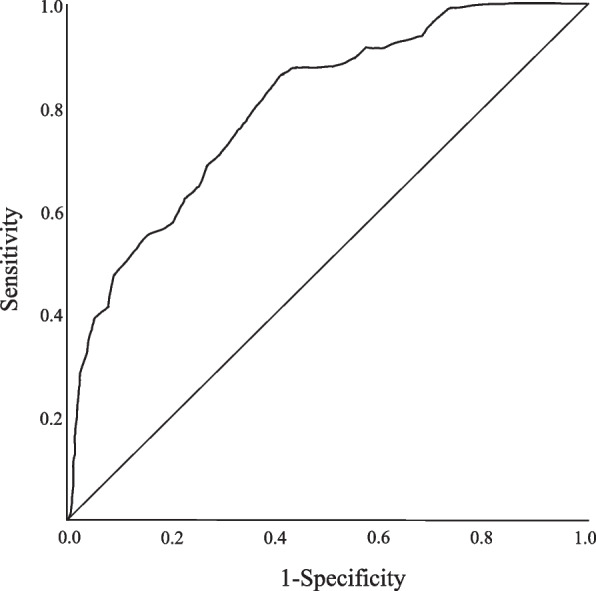
Results of receiver operating characteristic analysis. *Note.* Receiver operating characteristic analysis used the presence of hyperhidrosis symptoms as the independent variable and the scores of the anxiety specific to hyperhidrosis symptoms as the dependent variable. The sensitivity was 0.86, 1-Specificity was 0.41, and area under the curve was 0.80

To examine the discriminative accuracy and optimal cutoff values of the ASSHS, ROC curves were calculated (Fig. 2). A survival analysis using the total score of the 10-item ASSHS as the explanatory variable yielded a sensitivity of 0.86 and 1-specificity (false positive rate) of 0.41. Calculated from the ROC curve, the area under the curve (AUC) was 0.80 (95% Confidence Interval, 0.76–0.84), and the cutoff value for "anxiety about hyperhidrosis symptoms" based on the presence of diagnostic criteria for hyperhidrosis was 12 points.

## Discussion

This study tested the reliability and validity of our developed ASSHS. In addition, we compared each item based on the presence of hyperhidrosis symptoms, hyperhidrosis severity, and sweating site and calculated the cutoff scores of the ASSHS. As a result, 10 items with one factor of "anxiety specific to hyperhidrosis symptoms" were selected, and the internal consistency and retest reliability of the scale were found to be acceptable. Participants with hyperhidrosis symptoms had significantly higher ASSHS scores than did those without hyperhidrosis symptoms. Those with mild/moderate hyperhidrosis and those with severe hyperhidrosis had significantly higher ASSHS scores than did those without hyperhidrosis. Participants with palmar, plantar, axillary, and head/face hyperhidrosis had significantly higher ASSHS scores than did those without hyperhidrosis. Furthermore, a cutoff value of 12 points was suggested for the ASSHS, based on the presence or absence of diagnostic criteria for hyperhidrosis.

### Factorial validity and reliability of the ASSHS

This study examined the factor structure and reliability and validity of our developed ASSHS. Results of the exploratory factor analysis indicated that the questionnaire should include 10 items with one factor. Based on the confirmatory factor analysis, all indicators of model fit other than RMSEA had acceptable values, which suggests that this scale has sufficient validity. The Cronbach’s alpha coefficient was high for the total score of the ASSHS. Additionally, the correlation coefficient was also high after three weeks. These findings indicate that the ASSHS is a sufficiently reliable measurement scale.

### Criterion-related and construct validity of the ASSHS

A moderate positive correlation was observed between the ASSHS and HDSS, which measures the severity of hyperhidrosis and anxiety induced by sweating. This suggests that the ASSHS well reflects hyperhidrosis symptoms. Furthermore, its construct validity is adequate. Additionally, a positive correlation was observed between the ASSHS and STAI State anxiety, STAI Trait anxiety, and HADS Anxiety. This result supports its criterion-related validity. The DLQI and 36-Item Short Form Survey, which indicates skin-related QOL, are sometimes used to measure QOL among patients with hyperhidrosis. However, these methods include items that are not relevant to patients with hyperhidrosis. Hence, it was not possible to accurately understand the patients’ QOL [18, 27]. The ASSHS and STAI State anxiety, STAI Trait anxiety, and HADS Anxiety measure general anxiety and do not reflect hyperhidrosis symptoms, which could have resulted in weak correlations.

### Concomitant validity of the ASSHS

Hyperhidrosis symptomatic participants had significantly higher ASSHS scores than did those without. In a cross-sectional study on Japanese university students, anxiety induced by sweating was higher in symptomatic hyperhidrosis participants than in those without symptoms [15]. Patients with axillary hyperhidrosis were interviewed, and all reported embarrassment. Furthermore, 52% felt anxiety regarding sweating and concern regarding underarm odor [28]. Thus, previous studies reported that those with symptomatic hyperhidrosis had higher anxiety due to sweating, which is consistent with our results.

Those with mild/moderate hyperhidrosis and severe hyperhidrosis had significantly higher ASSHS scores than did those without. In an American cross-sectional study, 51% of those with mild to moderate hyperhidrosis symptoms experienced anxiety compared to 79% of those with severe symptoms [5]. In an international collaborative study from a combined Canadian and Chinese dermatology outpatient clinic, the prevalence of anxiety disorders was higher with greater severity of hyperhidrosis [10]. In the present study, the higher the severity of hyperhidrosis, the higher the ASSHS score, which was consistent with previous studies.

Those with palmar, plantar, axillary, and head/face hyperhidrosis had significantly higher ASSHS scores than did those without. A Brazilian cross-sectional study reported that patients with hyperhidrosis and anxiety symptoms were more affected in the axillary and head/face site than were those without symptoms [9]. Our results were inconsistent with previous studies, as there were no significant differences in scores for the ASSHS based on the site of sweating. Palmar and plantar hyperhidrosis have been reported to be predominantly caused by psychogenic sweating [29], while axillary and head/face hyperhidrosis were predominantly caused by thermogenic sweating [1, 30]. Therefore, palmar and plantar hyperhidrosis may be associated with higher ASSHS scores than are axillary and head/face hyperhidrosis. One reason for these different results could be the small sample sizes of those with plantar and head/face hyperhidrosis.

### Limitations and future issues

This study has some limitations. First, it was cross-sectional. Second, the survey was conducted only among students from one university, thus, the results cannot be generalized to all patients with hyperhidrosis. Future studies should conduct similar surveys for populations other than university students. Third, this study was based only on self-administered results and did not include a physician’s diagnosis of hyperhidrosis or assessment of symptom severity. The results should be interpreted with caution owing to the self-reported hyperhidrosis via the diagnostic criteria of Hornberger et al. (2004) [16] used in this study. Fourth, while treatment of hyperhidrosis has been reported to improve mental status [31, 32], we did not examine whether such treatment improves anxiety specific to hyperhidrosis symptoms; therefore, it is necessary to examine whether treatment for hyperhidrosis improves anxiety specific to hyperhidrosis symptoms. Fifth, as palmar and plantar hyperhidrosis are caused mainly by psychogenic sweating [29], it is possible that anxiety specific to hyperhidrosis symptoms is higher in palmar and plantar hyperhidrosis than in axillary and head/face hyperhidrosis, but the small sample size of participants with plantar hyperhidrosis and head/face hyperhidrosis did not allow for adequate examination of this issue. In the future, it will be necessary to increase the number of participants with plantar and head/face hyperhidrosis, who were underrepresented in this survey, and to conduct a detailed study of these cases. Sixth, the confirmatory factor analysis was based on the same participants whose data were used for the exploratory factor analysis. In the future, it will be necessary to conduct confirmatory factor analysis with a different sample.

## Conclusion

This study assessed the reliability and validity of our developed ASSHS. Consequently, 10 items with one factor of “anxiety specific to hyperhidrosis symptoms” were selected. This scale has sufficient reliability and validity as an instrument to measure anxiety specific to hyperhidrosis symptoms.

## Data Availability

The datasets used and/or analyzed during the current study are available from the corresponding author on reasonable request.

## Abbreviations

QOL: Quality of life
ASSHS: Anxiety scale specific to hyperhidrosis symptoms
HADS: Hospital Anxiety and Depression Scale
GAD-7: Generalized Anxiety Disorder
IAS-AD: Itch Anxiety Scale for Atopic Dermatitis
HDSS: Hyperhidrosis Disease Severity Scale
STAI: State-Trait Anxiety Inventory
DLQI: Dermatology Life Quality Index
χ^2^: Chi-square
GFI: Goodness-of-fit index
AGFI: Adjusted goodness-of-fit index
CFI: Comparative fit index
RMSEA: Root mean square error of approximation
ANOVA: Analysis of variance
ROC: Receiver operating characteristic
*df*: Degrees of freedom
AUC: Area under the curve

## References

[1] Fujimoto T, Yokozeki H, Nakazato Y, Murota H, Murayama N, Oshima Y (2023). Guidelines for the treatment of primary focal hyperhidrosis, revised 2023. Jpn J Dermatol..

[2] Stefaniak T, Tomaszewski KA, Proczko-Markuszewska M, Idestal A, Royton A, Abi-Khalil C (2013). Is subjective hyperhidrosis assessment sufficient enough? prevalence of hyperhidrosis among young Polish adults. J Dermatol..

[3] Liu Y, Bahar R, Kalia S, Huang RY, Phillips A, Su M (2016). Hyperhidrosis prevalence and demographical characteristics in dermatology outpatients in Shanghai and Vancouver. PLOS ONE..

[4] Shayesteh A, Janlert U, Brulin C, Boman J, Nylander E (2016). Prevalence and characteristics of hyperhidrosis in Sweden: a cross-sectional study in the general population. Dermatology..

[5] Doolittle J, Walker P, Mills T, Thurston J (2016). Hyperhidrosis: an update on prevalence and severity in the United States. Arch Dermatol Res..

[6] Augustin M, Radtke MA, Herberger K, Kornek T, Heigel H, Schaefer I (2013). Prevalence and disease burden of hyperhidrosis in the adult population. Dermatology..

[7] Fujimoto T, Inose Y, Nakamura H, Kikukawa Y (2023). Questionnaire-based epidemiological survey of primary focal hyperhidrosis and survey on current medical management of primary axillary hyperhidrosis in Japan. Arch Dermatol Res..

[8] Hamm H, Naumann MK, Kowalski JW, Kütt S, Kozma C, Teale C (2006). Primary focal hyperhidrosis: disease characteristics and functional impairment. Dermatology..

[9] Bragança GM, Lima SO, Pinto Neto AF, Marques LM, Melo EV, Reis FP (2014). Evaluation of anxiety and depression prevalence in patients with primary severe hyperhidrosis. An Bras Dermatol..

[10] Bahar R, Zhou P, Liu Y, Huang Y, Phillips A, Lee TK (2016). The prevalence of anxiety and depression in patients with or without hyperhidrosis (HH). J Am Acad Dermatol..

[11] Klein SZ, Hull M, Gillard KK, Peterson-Brandt J (2020). Treatment patterns, depression, and anxiety among US patients diagnosed with hyperhidrosis: a retrospective cohort study. Dermatol Ther (Heidelb)..

[12] Kristensen JK, Möller S, Vestergaard DG, Horsten HH, Swartling C, Bygum A (2020). Anxiety and depression in primary hyperhidrosis: an observational study of 95 consecutive Swedish outpatients. Acta Derm Venereol..

[13] Himachi M, Okajima I, Osawa K, Hashiro M, Sakano Y (2007). Development of the itch anxiety scale for atopic dermatitis (IAS-AD): reliability and validity. Jpn J Psychosom Med..

[14] Schneier FR, Heimberg RG, Liebowitz MR, Blanco C, Gorenstein LA (2012). Social anxiety and functional impairment in patients seeking surgical evaluation for hyperhidrosis. Compr Psychiatry..

[15] Ogawa S, Tayama J, Murota H, Kobayashi M, Kinoshita H, Nishino T (2023). Association of primary focal hyperhidrosis with anxiety induced by sweating: A cross-sectional study of Japanese university students focusing on the severity of hyperhidrosis and site of sweating. J Dermatol..

[16] Hornberger J, Grimes K, Naumann M, Glaser DA, Lowe NJ, Naver H (2004). Recognition, diagnosis, and treatment of primary focal hyperhidrosis. J Am Acad Dermatol..

[17] Strutton DR, Kowalski JW, Glaser DA, Stang PE (2004). US prevalence of hyperhidrosis and impact on individuals with axillary hyperhidrosis: results from a national survey. J Am Acad Dermatol..

[18] Kamudoni P, Mueller B, Salek MS (2015). The development and validation of a disease-specific quality of life measure in hyperhidrosis: the hyperhidrosis quality of life index (HidroQOL©). Qual Life Res..

[19] Solish N, Bertucci V, Dansereau A, Hong HC, Lynde C, Lupin M (2007). A comprehensive approach to the recognition, diagnosis, and severity-based treatment of focal hyperhidrosis: recommendations of the Canadian Hyperhidrosis Advisory Committee. Dermatol Surg..

[20] Fujimoto T, Kawahara K, Yokozeki H (2013). Epidemiological study and considerations of primary focal hyperhidrosis in Japan: from questionnaire analysis. J Dermatol..

[21] Abusailik MA, Mustafa SMB, Alzboun HM, Al-Issa HA, Oweis SW, Alshudeifat AY (2021). Primary hyperhidrosis: prevalence, severity, and impact on quality of life among Jordanian patients. Indian J Dermatol..

[22] Spielberger CD, Gorssuch RL, Lushene PR, Vagg PR, Jacobs GA. Manual for the State-Trait Anxiety Inventory. In: Palo Alto CA. USA: Consulting Psychologists Press; 1983.

[23] Hidano T, Fukuhara M, Iwawaki S, Soga S, Spielberger CD. State-Trait anxiety inventory-form JYZ. In: JITSUMUKYOIKU-SHUPPAN. Tokyo; 2000. (in Japanese).

[24] Zigmond AS, Snaith RP (1983). The hospital anxiety and depression scale. Acta Psychiatr Scand..

[25] Hatta H, Higashi A, Yashiro H, Ozawa K, Hayashi K, Kiyota K (1998). A validation of the hospital anxiety and depression scale. Jpn J Psychosom Med..

[26] Takahashi N, Suzukamo Y, Nakamura M, Miyachi Y, Green J, Ohya Y (2006). Japanese version of the Dermatology Life Quality Index: validity and reliability in patients with acne. Health Qual Life Outcomes. Japanese version..

[27] Young O, Neary P, Keaveny TV, Mehigan D, Sheehan S (2003). Evaluation of the impact of transthoracic endoscopic sympathectomy on patients with palmar hyperhydrosis. Eur J Vasc Endovasc Surg..

[28] Nelson LM, DiBenedetti D, Pariser DM, Glaser DA, Hebert AA, Hofland H (2019). Development and validation of the axillary sweating daily diary: a patient-reported outcome measure to assess axillary sweating severity. J Patient Rep Outcomes..

[29] Sato K, Kang WH, Saga K, Sato KT (1989). Biology of sweat glands and their disorders. II. Disorders of sweat gland function. J Am Acad Dermatol..

[30] Sammons JE, Khachemoune A (2017). Axillary hyperhidrosis: a focused review. J Dermatolog Treat..

[31] Connor KM, Cook JL, Davidson JR (2006). Botulinum toxin treatment of social anxiety disorder with hyperhidrosis: a placebo-controlled double-blind trial. J Clin Psychiatry..

[32] Kamikava DYF, Wolosker N, Silva MFAD, Campos JRM, Puech-Leão P (2021). Symptoms of anxiety and depression in patients with primary hyperhidrosis and its association with the result of clinical treatment with oxybutynin. Clinics (Sao Paulo)..

